# Genetic Programming by Nitric Oxide-Sensing Gene Switch System in Tumor-Targeting Bacteria

**DOI:** 10.3390/bios13020266

**Published:** 2023-02-13

**Authors:** Yeshan Qin, Sung-Hwan You, Ying Zhang, Akhil Venu, Yeongjin Hong, Jung-Joon Min

**Affiliations:** 1 Department of Molecular Medicine, Chonnam National University Graduate School, Gwangju 61469, Republic of Korea; 2 Institute for Molecular Imaging and Theranostics, Hwasun Hospital, Chonnam National University Medical School, Gwangju 58128, Republic of Korea; 3 Department of Microbiology, Chonnam National University Medical School, Gwangju 58128, Republic of Korea; 4 Department of Nuclear Medicine, Chonnam National University Medical School, Gwangju 61469, Republic of Korea

**Keywords:** synthetic biology, nitric oxide, DNA recombinase, DNA switch, oncolytic bacteria

## Abstract

Recent progress in synthetic biology has enabled bacteria to respond to specific disease signals to perform diagnostic and/or therapeutic tasks. *Salmonella enterica* subsp. *enterica* serovar Typhimurium (*S.* Typhimurium) colonization of tumors results in increases in nitric oxide (NO) levels, suggesting that NO may act as a candidate inducer of tumor-specific gene expression. The present study describes a NO-sensing gene switch system for triggering tumor-specific gene expression in an attenuated strain of *S.* Typhimurium. The genetic circuit was designed to sense NO via NorR, thus initiating the expression of FimE DNA recombinase. This was found to lead sequentially to the unidirectional inversion of a promoter region (*fimS*), which induced the expression of target genes. Target gene expression in bacteria transformed with the NO-sensing switch system was triggered in the presence of a chemical source of NO, diethylenetriamine/nitric oxide (DETA/NO) in vitro. In vivo results revealed that the gene expression is tumor-targeted, and specific to NO generated by inducible nitric oxide synthase (iNOS) after *S.* Typhimurium colonization. These results showed that NO was a promising inducer to finely tune the expression of target genes carried by tumor-targeting bacteria.

## 1. Introduction

Synthetic biology is a multidiscipline that artificially engineers genetic circuits to display specific biological behaviors in cells [1,2,3]. These genetically engineered bacterial cells act as novel therapeutics, with advantages when compared with small molecules or biologic agents. The localization of these cells can be better controlled, their retention times can be prolonged, and their ability to release a sufficient dose of drug in response to specific biomarkers can be better regulated [4]. Several microorganisms have been engineered for diagnostic or therapeutic purpose. Engineered *Escherichia coli* (*E. coli*) have been developed to sense reactive oxygen species (ROS) indicator, tetrathionate, to detect gut inflammation [5], and engineered *Salmonella enterica* subsp. *enterica* serovar Typhimurium (*S.* Typhimurium) were developed to deliver anticancer proteins to tumor tissues, with genes placed under the control of promoters that respond to specific signals [3].

Since streptococcal microorganisms were first injected into cancer patients to suppress malignant tumors in the 19th century [6], the anticancer activities of various bacteria have been explored [7]. *S.* Typhimurium has shown high tumor-targeting specificity, the ability to penetrate deep into tumor tissues, and robust anticancer activity. An attenuated *S.* Typhimurium defective in ppGpp biosynthesis (*SL*. ΔppGpp) showed tumor: normal organ bacterial ratio of over 10,000:1, with bacterial cells reaching 1 × 10^7^ CFU/g in tumor 3 days after intravenous injection [8,9], and cause various anticancer immune responses, including recruiting immune cells and secretion of proinflammatory cytokines, such as IFN-γ, TNF-α, and IL-1β [10,11,12,13]. *S.* Typhimurium in tumors has also been shown to upregulate the expression of inducible nitric oxide synthase (iNOS) [14,15,16], an enzyme converting _L_-arginine into _L_-citrulline and nitric oxide (NO) that can be regulated by proinflammatory cytokines and bacterial lipopolysaccharide (LPS) [17,18,19,20]. This upregulation of iNOS upon *S.* Typhimurium infection increases NO generation up to 1000-fold to a micromolar range [10,20,21,22]. These findings suggest that NO may act as an endogenous inducer of tumor-specific expression of genes carried by bacteria.

NorR is a σ^54^-dependent regulatory protein in *E. coli* that sense NO to activate NO detoxification genes (*norV* and *norW*, encoding *E. coli* flavorubredoxin and its associated oxidoreductase, respectively) via its binding to the promoter for *norV* (P*_norV_*) in the *norR-norVW* intergenic region [23]. NorR consists of three domains, a GAF (cGMP-specific and -stimulated phosphodiesterases, Anabaena adenylate cyclases, and *E. coli* FhlA) domain at the N-terminus, an AAA+ (ATPase associated with various cellular activities) domain at the center, and a DNA-binding domain at the C-terminus [24]. In the absence of NO, NorR binds via its DNA-binding domain to three-repeated enhancer sequences in the P*_norV_* promoter, and its GAF domain negatively regulates the activity of its AAA+ domain by blocking the binding of σ^54^, a subunit of RNA polymerase. This prevents the transcription of genes downstream of the P*_norV_* promoter. In the presence of NO, the GAF domain binds to NO, inducing the AAA+ domain to bind σ^54^, resulting in transcription from the P*_norV_* promoter [25,26].

Uropathogenic *E. coli* possesses the fimbriae phase 1 variation system, a process that controls gene expression through an ON/OFF switch that is mediated by site-specific DNA recombination [27]. The phase variation of fimbriae occurs through the inversion of a 314 bp DNA element (*fimS*), which contains the promoter to *fimA* [28,29,30]. Two site-specific recombinases, FimB and FimE, are involved in *fimS* inversion by functioning on two inverted repeated sequences, called Inverted Repeat Right (IRR) and Inverted Repeat Left (IRL), respectively, which flank *fimS*. Since the binding affinities of FimE and FimB towards IRR and IRL differ, FimB can invert *fimS* in both directions, whereas FimE can only change the orientation of *fimS* when it faces the IRR; therefore, the switch caused by FimE is unidirectional, permanent, and inheritable [31,32,33]. 

The present study describes the in vivo NO-triggered tumor-targeting gene delivery by a NO-sensing gene switch system with two plasmids. In one plasmid, the *fimE* gene was placed under the control of the P*_norV_* promoter, whereas in the second plasmid, target genes of interest (GOIs) were fused with the *fimS* fragment in the opposite direction, and NorR is constitutively expressed under P*_lacIq_* promoter. Switch activation was observed in tumor-targeting bacteria with the NO-sensing system after its colonization in tumor in vivo.

## 2. Materials and Methods

### 2.1. Bacterial Strains and Culture Conditions

*E. coli* DH10-beta (NEB, #C3019H) was used for cloning and amplification of plasmids. The plasmids were constructed by T4 DNA ligase (Thermo Fisher Scientific, Waltham, MA, USA) after digestion with restriction enzymes (NEB, Ipswich, MA, USA) or using a Gibson assembly kit (NEB, USA). KOD Plus Neo DNA polymerase (Toyobo, Japan) was used for polymerase chain reaction (PCR) amplification. Oligonucleotides were chemically synthesized (Macrogen, Seoul, Korea). The attenuated strain of *S.* Typhimurium SHJ2037 (*relA::cat*, *spoT::kan*), renamed *SL*. ΔppGpp in this study [34], was transformed with plasmids by 2.5 kV electroporation (Bio-Rad, Des Planies, IL, USA). These bacteria were grown in Luria-Bertani (LB) broth or on LB agar plates supplemented with the appropriate antibiotics at 37 °C. Transformed bacterial cells were stored at −80 °C in 25% glycerol before in vitro and in vivo assessments of the NO-sensing gene switch system.

### 2.2. Construction of Plasmids for NO-Sensing Gene Switch System

NIA3, a plasmid containing the NO-sensing elements (*P_norV_*, *fimE*, *P_lacIq_*-*NorR*, and *fimS*), was the kind gift of Dr. M. Shapiro (California Institute of Technology, Pasadena, CA, USA). The plasmids pTD103-sfGFP (#48885, Addgene) and pTU2S-a (#74088, Addgene), containing fragments of the *super-folder green fluorescent protein* (*sfGFP*) gene and the p15A origin of replication (*ori*), respectively, were gifts from Dr. J. Hasty (University of California, San Diego, CA, USA) and Dr. P. Freemont (Imperial College London, UK), respectively. pBAD-Rluc8 with pBR322 *ori* was described previously [35,36]. The gene fragments were amplified with PCR using KOD Plus Neo DNA polymerase.

The *P_norV_* fragment in the NIA3 plasmid was amplified using the primers P*_norV_*-F (5′-CG GAA TAT ATC CCC TAG GGC GCT GAA AAC GAT CCT G-3′) and P*_norV_*-R (5′-TAG CAA CCT CAA TTT ATT CAG CGT GTT C-3′). The *fimE* fragment lacking a ribosomal binding site (RBS) in the NIA3 plasmid was amplified using the primers noRBS-FimE-F (5′-GAA TAA ATT GAG GTT GCT AAT GGT GAG TAA ACG TCG TTA TCT TAC C-3′) and FimE-R (5′-ATC CGC CGC CCT AGA CCT AGG GAC TAG TTC AAA CCT CTT CTC TTT TTA ATT TTT C-3′). The two PCR fragments were ligated using a Gibson assembly kit, with the resulting *P_norV_::fimE* fragment ligated into the *Avr*II-cut pTU2S-a plasmid. The plasmid containing p15A *ori*, called pFimE, contained a *fimE* gene driven by the P*_norV_* promoter and a chloramphenicol resistance (Cm^R^) gene.

The NorR gene fragment with P*_lacIq_* promoter (*P_lacIq_::norR*) in the NIA3 plasmid was amplified using the primers NOR-F (5′-TTG CTG GCC TTT TGC TCA GAC ACC ATC GAA TGG TGC-3′) and NOR-R (5′-AAC GCA GGA AAG AAC ATG TCG CAG AAA GGC CCA CC-3′) against NIA3, and ligated into *Pci*I-cut pBAD-Rluc8 using a Gibson assembly kit. The resulting plasmid, named pBR-NOR, contained an ampicillin resistance (Amp^R^) gene. 

The *fimS* (IRL-*fimS*-IRR) fragment in the NIA3 plasmid was amplified using the primers *fimS* block-F (5′-AAA AAC ATG TCG CAG CCA TGA CCC AGT C-3′) and *fimS* block-R (5′-GGT ACC TTT CTC CTC TTT AAT ACA AGA ACA ATT GGG GCC CAT TTT G-3′). The *rluc8* fragment encoding *Renilla* luciferase variant 8 with a strong RBS sequence (AAAGAGGAGAAA) in the plasmid pBAD-Rluc8 was amplified using the primers Rluc8-F (5′-AAA GAG GAG AAA GGT ACC ATG GCT TCC AAG GTG TAC GAC-3′) and Rluc8-R (5′-TGC GGT CGA CCT GCT CGT TCT TCA GCA CG-3′). These two fragments were ligated by Gibson assembly kit and named *fimS::rluc8*. The *fimS::rluc8* fragment and pBR-NOR were digested with *Sal*I and *Pci*I and ligated by T4 DNA ligase. The resulting plasmid was named pSRluc8.

The *sfGFP* gene fragment with a strong RBS sequence in the plasmid pTD103-sfGFP was amplified using the primers sfGFP-F (5′-AAA GAG GAG AAA GGT ACC ATG AGC-3′) and sfGFP-R (5′-AGC TTT GTT TAA ACT CAT TTG TAG AGC TCA TCC ATG CCA TG-3′), with this fragment ligated with the above-described *fimS* fragment using a Gibson assembly kit. This fragment, named *fimS::sfGFP*, was digested with *Pci*I and *Pme*I, and ligated by T4 DNA ligase into pBR-NOR digested with same restriction enzymes. The resulting plasmid was named pSGFP.

All plasmids were sequenced (Macrogen, Korea). The resulted plasmids and *SL.* ΔppGpp transformants were listed in Table S1. 

### 2.3. Analysis of Nitric Oxide (NO)-Sensing Gene Switching Events

*SL.* pNASR and *SL.* pRluc8 were cultured at 37 °C overnight with shaking at 200 rpm in LB broth supplemented with ampicillin (100 μg/mL) and/or chloramphenicol (34 μg/mL). These cultures were diluted 1:100 into 3 mL of fresh LB broth and further cultured to mid-log phase, defined as an optical density at 600 nm (OD_600_) of 0.5. The bacterial cultures were treated with various concentrations (0–1000 μM) of diethylenetriamine/nitric oxide (DETA/NO, Abcam, Cambridge, UK) and incubated with shaking at 37 °C. After 16 h, the bacterial pellets were harvested by centrifugation and washed with phosphate-buffered saline (PBS). 

To measure the Rluc8 activity of the activated switch, 10 μL aliquots of 40 μg/mL coelenterazine, a luciferase substrate, were added to pellets resuspended in 200 μL PBS in a 96-well plate. The bioluminescence signals were measured with OrionL Microplate luminometer (Titertek Berthold, Pforzheim, Germany) and normalized to OD_600_. The bioluminescence images of each well were taken with an IVIS Lumina imaging system (PerkinElmer, Waltham, MA, USA). The levels of expression of Rluc8 protein were determined by western blotting using primary anti-*Renilla* luciferase antibody (Abcam, Cambridge, UK) and secondary horseradish peroxidase (HRP)-conjugated antibody. DnaK was used as control and detected with anti-DnaK antibody (Enzo Life Science, Farmingdale, NY, USA).

To measure the activated switch percentage under various concentrations of DETA/NO, *SL.* pNASGFP were treated with DETA/NO for 16 h, washed, and resuspended in PBS. The bacterial cells were serially diluted with PBS and spread onto LB agar plates containing antibiotics. After culture for overnight, the plates were placed under UV light to count green-fluorescent colonies. The percentages of green-fluorescent colonies relative to total colonies were calculated. 

### 2.4. PCR Analysis of Switch Inversion

*SL.* pNASR were treated with the indicated concentrations of DETA/NO, and 100 μL aliquots were killed at 100 °C for 5 min. The switch events of *fimS* were detected by PCR using the primers, *fimS* (5′-GTC GAG CCA CAG AAA CGT TAG CTT TAC ATA TAG-3′) and *rluc8* (5′-TCA CTG CTC GTT CTT CAG CAC GC-3′), with the expected size of the PCR band of the switched *fimS::rluc8* gene being ~1.1 kb. Each reaction contained 1 μL of each primer (10 pmol) and 1 μL of heat-killed bacterial cells in a total reaction volume of 20 μL, with the amplification protocol consisting of 30 cycles. 

### 2.5. Measurement of Time-Dependent Switch Activation

*SL.* pNASR were treated with 100 μM DETA/NO and further cultured at 37 °C. Aliquots were collected after 0–8 h in culture, with the resulting bacterial pellets resuspended in PBS. Rluc8 activity was measured by determining bioluminescence intensities in the presence of coelenterazine, as described above. The switch events were also analyzed by PCR using *fimS* and *rluc8* primers.

### 2.6. Switch Inheritance Verification

*SL*. pNASR were cultured in the presence of 100 μM DETA/NO for 16 h, diluted 100-fold in 3 mL of fresh media with antibiotics and without DETA/NO, and further cultured for 72 h. Rluc8 activity at the indicated times was measured by determining bioluminescence intensities in the presence of coelenterazine. 

### 2.7. Analysis of In Vivo Switch Activation via Bioluminescence Imaging of Rluc8 Expression

All animal experiments and euthanasia procedures were performed in accordance with protocols approved by the Animal Research Committee of Chonnam National University, Korea. Female Balb/C mice aged 6 weeks were purchased from Orient (Korea) and anesthetized with 2% isoflurane (Hana Pharm, Seoul, Korea). Tumors were generated by subcutaneous implantation of 5 × 10^5^ CT-26 colon carcinoma cells (CRL-2638, American Type Culture Collection, Manassas, VA, USA) in 100 μL PBS into the right flank of each mouse. When the tumor sizes reached about 150 mm^3^, *SL*. pNASR (1 × 10^7^ in 100 μL PBS) were injected intravenously into each mouse. The mice were subjected to in vivo bioluminescence imaging with coelenterazine (20 μg in 100 μL PBS, i.v.) on the indicated days after bacterial injection using an IVIS Lumina S5 imaging system (PerkinElmer, Waltham, MA, USA). The levels of bioluminescence in the tumor region were measured as corresponding photon signals.

### 2.8. In Vivo NO-Specific Switch Activation via Inducible Nitric Oxide Synthase (iNOS) Activity Inhibition

To assess the effects of inhibition of iNOS activity, an iNOS inhibitor, 1400 W dihydrochloride (200 μg per injection), was intraperitoneally injected into CT-26 tumor-bearing mice every day, beginning 3 days before bacterial injection to the end of the experiment. Bioluminescence images were obtained on the indicated days after *SL.* pNASR injection. The levels of bioluminescence in the tumor region were measured as corresponding photon signals.

### 2.9. Measurement of iNOS Expression in Tumors Treated with Bacteria Bearing the NO-Sensing Gene Switch System

Mice treated with *SL*. pNASR or PBS (control) were sacrificed on the indicated days. The tumors were removed, weighed, and homogenized in a 4-fold volume of ice-cold NP40 lysis buffer containing 1x protease inhibitor cocktail (genDEPOT, Barker, TX, USA). After incubation for 1 h on ice, the samples were centrifuged at 13,000 rpm for 30 min at 4 °C and passed through a 0.2 μm filter, with the supernatants stored at −80 °C prior to further analysis. The supernatants were thawed on ice, and their total protein concentrations were measured using a BCA assay kit (Thermo Fisher Scientific, Waltham, MA, USA). The protein concentration of each sample was adjusted to 1 μg/μL with NP40 lysis buffer containing 1x protease inhibitor cocktail. Aliquots containing 10 μg protein were separated on 8% SDS-PAGE gels and transferred to PVDF membranes (GE Healthcare Life Sciences, Chicago, IL, USA). The membranes were incubated overnight at 4 ℃ with mouse anti-iNOS monoclonal antibody (1:1000, Abcam, Cambridge, UK) or rabbit anti-β-actin antibody (1:1000, SantaCruz, Dallas, TX, USA), followed by incubation with HRP-conjugated anti-mouse Ig (1:1000, Invitrogen, USA) or anti-rabbit Ig (1:500, Thermo Fisher Scientific, Waltham, MA, USA) secondary antibodies for 1 h at room temperature. The resulting bands were photographed with ChemiDocTM XRS+ machine (Bio-Rad, Des Planies, IL, USA). The intensities of iNOS bands were measured by ImageJ software and normalized against those of β-actin bands.

### 2.10. Measurement of Proinflammatory Cytokines by Enzyme-Linked Immunosorbent Assays (ELISA)

Concentrations of the cytokines interferon (IFN)-γ, interleukin (IL)-1β, and tumor necrosis factor (TNF)-α were measured by corresponding ELISA kits (Thermo Fisher Scientific, Waltham, MA, USA), according to the manufacturer’s protocols. 

### 2.11. Immunofluorescence Staining of sfGFP in Tumors Treated with Bacteria Carrying the NO-Sensing Gene Switch System

Mice treated with *SL.* pNASGFP or PBS (control) were sacrificed on the indicated days after bacterial injection. Tumor tissues were embedded in optimal cutting temperature (OCT) compound (Thermo Fisher Scientific, Waltham, MA, USA) and stored at −80 °C. Frozen tumor sections (5 μm thick) were made with a cryomicrotome (Thermo Fisher Scientific, Waltham, MA, USA) and mounted onto glass slides. The slides were blocked with 5 % BSA at room temperature for 1 h, and then incubated overnight at 4 °C with mouse anti-iNOS monoclonal antibody (1:100, Invitrogen, Waltham, MA, USA). After three washes with PBS containing 0.5% Tween-20 (PBS-T), the slides were incubated with Alexa 555-conjugated anti-Rat Ig antibody (1:200, Thermo Fisher Scientific, Waltham, MA, USA) in the dark for 1 h. After washing with PBS-T, the slides were mounted in ProLong antifade mounting solution with 4′,6-diamidino-2-phenylindole dye (DAPI, Invitrogen, Waltham, MA, USA) and sealed with nail polish. The fluorescence signals of sfGFP (green), iNOS (red), and DAPI (blue) were detected using an LSM510 fluorescence microscope (Zeiss, Jena, Germany), and processed using LSM image software.

### 2.12. Statistical Analysis

Statistical analysis was done with Student’s *t*-tests. A *p*-value < 0.05 was considered statistically significant. All data are expressed as mean ± SEM.

## 3. Results 

### 3.1. Study Design and Construction of Nitric Oxide (NO)-Sensing Gene Switch System

**Figure 1:**
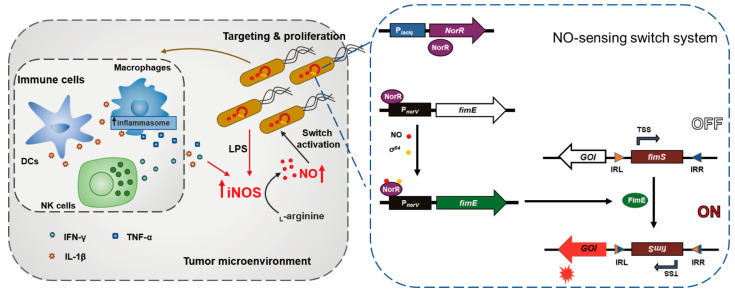
Conceptual illustration of this study. Colonization by and proliferation of *SL*. ΔppGpp in tumor regions can lead to recruitment and activation of innate immune cells, such as macrophages, dendritic cells (DCs), and natural killer (NK) cells. Proinflammatory cytokines secreted by immune cells, including interferon (IFN)-γ, tumor necrosis factor (TNF)-α, and interleukin (IL)-1β, as well as bacterial components, such as lipopolysaccharide (LPS), can upregulate the expression of iNOS, thus generating high levels of NO. NO binding to NorR can recruit σ^54^ and release the suppression by NorR of the *norVW* promoter P*_norV_*, resulting in transcription of FimE recombinase. FimE can function on the inverted repeat left (IRL) and right (IRR) regions flanking *fimS*, which contains a promoter region, leading to the inversion of *fimS* and expression of the gene of interest (GOI). TSS, transcription start site.

The NO-sensing gene switch system was designed as illustrated (Figure 1). Briefly, the elevated NO generated by the increase in inducible nitric oxide synthase (iNOS) after bacterial colonization was used to trigger gene expression from the NO-sensing switch system in *SL*. ΔppGpp (an attenuated *S.* Typhimurium defective in ppGpp biosynthesis). The switch system was constructed such that when *fimS* faced IRR, no gene was expressed, defined as the OFF state. When NO triggered the expression of FimE, leading to the reorientation of *fimS*, the GOI was expressed, defined as the ON-state.

**Figure 2:**
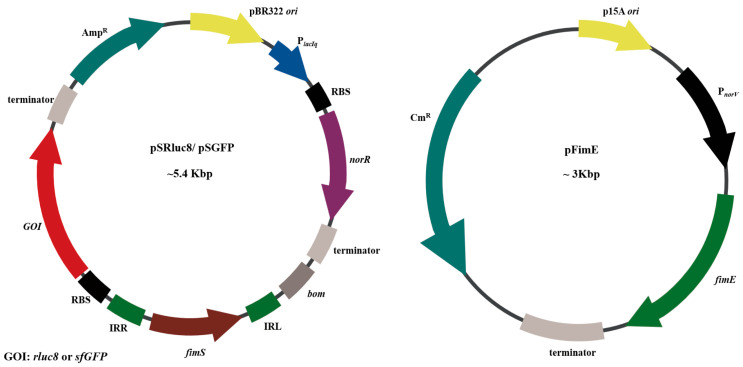
Plasmid construction. pSRluc8/pSGFP represents the plasmid containing the *fimS* switch block (IRR-*fimS*-IRL-RBS-GOI), which also constitutively expresses the NO sensor NorR, resulting in the repression of P*_norV_*. pFimE represents the plasmid for NO-induced FimE expression under the control of P*_norV_*, which is suppressed by NorR in the presence of low or no NO. *ori*, replication origin; RBS, ribosome binding site; IRL, inverted repeat left; IRR, inverted repeat right; Amp^R^, ampicillin resistance gene; Cm^R^, chloramphenicol resistance gene.

The NO-sensing gene switch system was constructed with two plasmids (Figure 2). One plasmid, with a high-copy pBR322 replication origin (*ori*), contained a *fim* switch block (IRR-*fimS*-IRL-GOI), with either *Renilla* luciferase variant 8 (*rluc8*) [36,37] or *super-folder green fluorescent protein* (*sfGFP*) [38] acting as the reporter gene, with strong ribosome binding site (RBS). The *fimS* gene containing a promoter region was located upstream of GOI in the reverse direction, with GOI expression triggered after the reorientation of *fimS*. This plasmid also contained the *norR* gene with a constitutive P*_lacIq_* promoter and the same RBS. This combination has been used to suppress additional copies of P*_norV_* on exogenous plasmids [33]. The resulting plasmids containing *rluc8* and *sfGFP* were named pSRluc8 and pSGFP, respectively. 

Another plasmid, pFimE, was responsible for the NO-driven expression of FimE recombinase. Leaky expression of FimE recombinase has often led to gene switching, even under non-sensing conditions [39], indicating that a small amount of FimE was sufficient to drive the reorientation of the *fimS* gene fragment. In the pFimE plasmid, the fimE gene without a typical RBS was under the control of the P*_norV_* promoter for low level of expression and was inserted into pTU2S-a with a low-copy p15A *ori* [40]. 

### 3.2. Characterization of Bacteria Transformed with the NO-Sensing Switch System

To characterize the NO-responsive properties of the constructed switch system, diethylenetriamine/nitric oxide (DETA/NO) was used as an in vitro NO source. 

**Figure 3:**
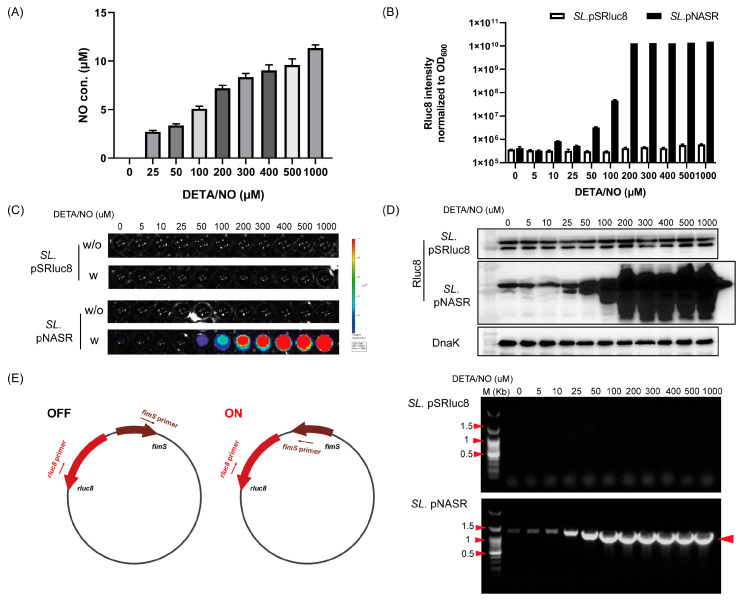
Dose responsive analysis of the NO-sensing switch system. (**A**) NO release by different concentrations of DETA/NO in bacterial culture medium after incubation at 37 °C for 16 h. (**B**) Rluc8 intensity in *SL*. pNASR and *SL*. pSRluc8 cultured in the presence of indicated concentrations of DETA/NO for 16 h at 37 °C. Rluc8 intensity was measured with 0.2 μg of coelenterazine and normalized to the number of bacterial cells (OD_600_). (**C**) Bioluminescence images of *SL*. pSRluc8 (upper panel) and *SL*. pNASR (bottom panel) from (**B**). *w*/*o*, without coelenterazine; *w*, with coelenterazine. (**D**) Western blot analysis of Rluc8 expression in bacterial samples from (**B**) using anti-*Renilla* luciferase antibody (upper panel). Rluc8 expression was normalized to DnaK expression, as detected with anti-DnaK antibody (bottom panel). (**E**) Switch activation verified by PCR amplification. Left, illustration of ON-state primer designation; right, PCR results using ON-state primer set to confirm switch activation in bacterial cells from (**B**). Bands appeared when *fimS* was switched and in the same direction as *rluc8* (~1.1 Kbp, arrow).

NO levels released by DEAT/NO were measured in bacterial culture media after incubation for 16 h at 37 °C (Figure 3A). NO levels ranged from ~3 μM in the presence of 25 μM DEAT/NO to ~12 μM in the presence of 1000 μM DEAT/NO. 

The NO sensitivity of the switch system was evaluated by incubating *SL*. pNASR with various concentrations (0–1000 μM) of DEAT/NO, with same treated *SL*. pSRluc8 being the control (Figure 3B–E). Switch activation was indicated by Rluc8 expression and Rluc8 intensity was measured (Figure 3B,C). Bioluminescence signal was significantly higher at 50 μM DEAT/NO, increased dose-dependently, and reached a maximum at 200 μM DEAT/NO. By contrast, *SL*. pRluc8 transformed with pRluc8 alone did not show any signal, even at 1000 μM DEAT/NO. These results were consistent with that of western blot analysis of Rluc8 expression (Figure 3D). The switch of *fimS* was also detected by PCR analysis using an ON-state primer set (Figure 3E). No band was detected in *SL*. pSRluc8, even at 1000 μM DEAT/NO. In *SL*. pNASR, negligible bands were detected at no or low DETA/NO concentrations, which were likely due to the leaky expression of FimE and NO produced by bacteria themselves. However, the band intensities were markedly increased at 25 μM and higher DETA/NO concentrations. These findings indicated that activation of the NO-sensing switch system was dependent on elevated NO, that the switch was activated at 25 μM DETA/NO, and that it reached a detectable level at 50 μM.

The percentage of switch activated at various concentrations of DETA/NO was investigated using *SL*. pNASGFP carrying a *sfGFP* gene. (Figure S1). The percentages of fluorescent colonies were dependent on DETA/NO concentrations, with 20%, 70%, and 100% fluorescent colonies detected at DETA/NO concentrations of 100, 200, and 1000 μM, respectively. These results suggested that the phenotype of this NO-sensing switch system can be detected at 100 μM DETA/NO (~5 μM NO).

The timing of the gene switching event in the presence of DETA/NO was determined by incubating *SL*. pNASR with 100 μM DETA/NO and measuring Rluc8 intensity by PCR analysis (Figure S2). In the presence of DETA/NO, strong Rluc8 intensity was detected in *SL*. pNASR after incubating for 30 min, consistent with the band detected by PCR test. These results indicated that NO induction of the switch event was very rapid.

**Figure 4:**
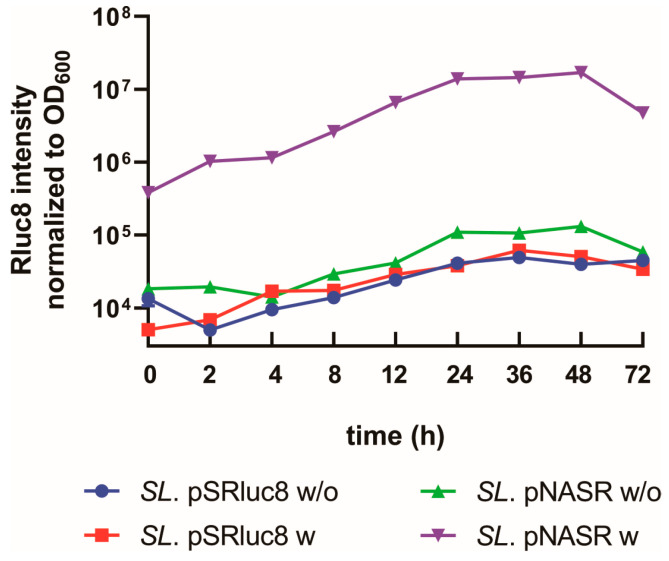
Switch inheritance and permanency. *SL*. pSRluc8 and *SL*. pNASR were incubated overnight without (*w*/*o*) or with (*w*, 100 μM) DETA/NO, and diluted 1:100 in fresh LB media without DETA/NO. Rluc8 intensity was measured at the indicated time points after removal of DETA/NO, and was normalized to the number of bacterial cells (OD_600_). Each point represents the mean ± SEM of triplicate determinations.

FimE catalyzes the unidirectional switch of *fimS*, as it shows different binding affinities towards IRL and IRR, and is only able to invert *fimS* when it faces IRR. Thus, the switch inversion induced by FimE is both inheritable and permanent [27,33]. To determine the switch inheritance and permanency of the NO-sensing system, *SL*. pNASR was incubated overnight with 100 μM DETA/NO, followed by culture for an additional 72 h in the absence of DETA/NO (Figure 4). Rluc8 intensity was measured at the indicated time points. In the absence of DETA/NO pretreatment, the bioluminescence intensity in *SL*. pNASR was similar to that of control *SL*. pSRluc8 (0 h). DETA/NO pretreatment increased the Rluc8 intensity in *SL*. pNASR at 0 h by about 200-fold compared with other groups, indicating that the switch had been activated. Rluc8 intensity gradually increased, being more than 10-fold higher at 48 h than at 0 h but was lower at 72 h, which was thought to be due to bacterial cells being in the death phase of the growth curve. This result indicated that the switched gene was maintained permanently in the ON-state during bacterial division and plasmid replication, even in the absence of further NO stimulation after activation. 

### 3.3. In Vivo Tumor-Specific Gene Delivery by Bacteria with NO-Sensing Gene Switch System

**Figure 5:**
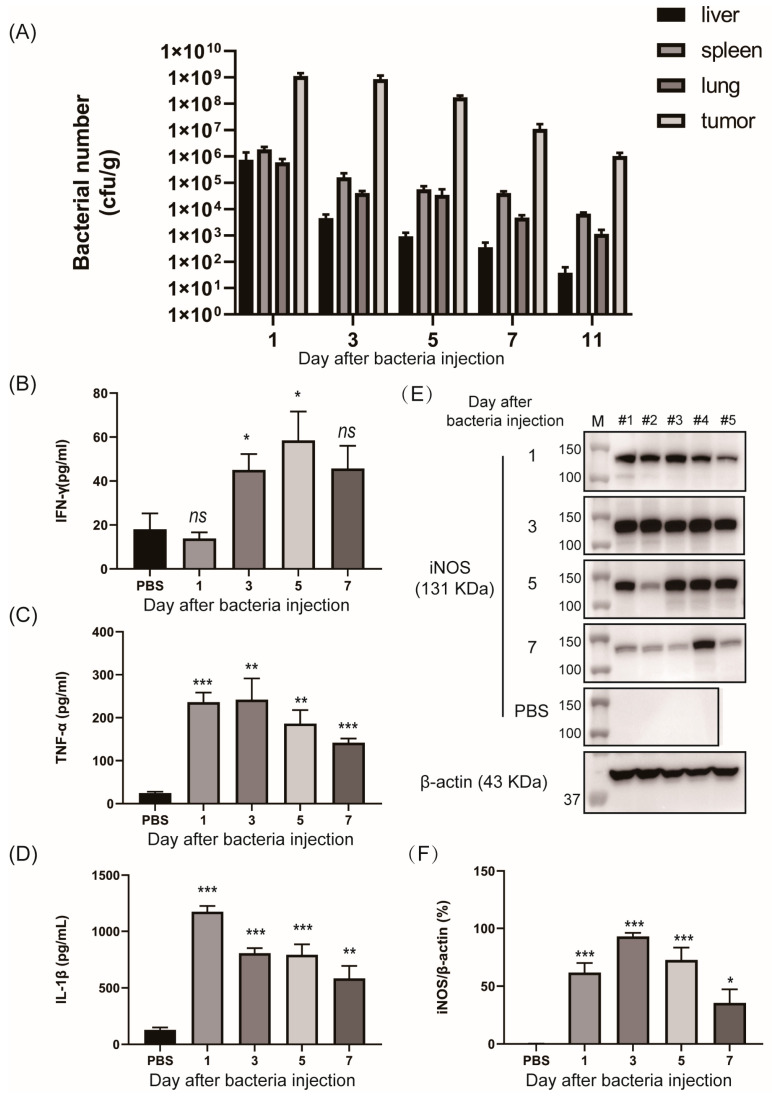
In vivo assessment of tumor microenvironment modulation after bacterial colonization. CT26-bearing mice inoculated with 1 × 10^7^ CFUs of *SL*. pNASR (*n* = 5) or PBS (*n* = 4) were sacrificed on days 1, 3, 5, and 7. (**A**) Numbers of bacterial cells colonizing normal organs (liver, spleen, and lungs) and tumor tissues. (**B**–**D**) Tumor concentrations of the proinflammatory cytokines (**B**) IFN-γ, (**C**) TNF-α, and (**D**) IL-1β on indicated days post-injection. (**E**) Western blot determination of iNOS expression in tumors. (**F**) Quantification of iNOS expression level normalized to β-actin (%). Error bars represent the mean ± SEM. * *p* < 0.05, ** *p* < 0.01, and *** *p* < 0.001; ns, not significant, as determined by Student’s *t*-tests.

To assess whether *SL*. ΔppGpp bearing the NO-sensing gene switch system specifically colonized and induced immune response in tumors in vivo (Figure 5), *SL*. pNASR was intravenously injected into CT26 tumor-bearing mice. The numbers of colonizing bacterial cells on day 3 were about 10,000-fold higher in tumors (~10^9^ colony forming unit (CFU)/g) than in normal organs, such as the liver, spleen, and lungs (~10^5^ CFU/g) (Figure 5A). The numbers of bacterial cells in tumors gradually decreased over time, but remained higher in tumors than in other organs until day 11. The immune responses induced by bacterial cells colonizing in tumors were evaluated by measuring the levels of the proinflammatory cytokines, including IFN-γ, TNF-α, and IL-1β, in the tumor tissues (Figure 5B–D). The levels of TNF-α and IL-1β were significantly higher, beginning on day 1, and the levels of IFN-γ were significantly higher, beginning on day 3, in bacteria-treated mice than in the PBS group, similar to previous reports [11,12]. 

The expression of iNOS can be upregulated in response to bacterial LPS and proinflammatory cytokines [15,20]. This was confirmed by measuring iNOS expression levels in tumors treated with *SL*. pNASR (Figure 5E,F). iNOS protein in tumors was found to be expressed on day 1, maximized on day 3 with up to a 500-fold increase, and decreased on day 7. These results confirmed that iNOS, an enzyme generating high amounts of NO, was highly and rapidly upregulated after bacterial colonization of tumors. 

**Figure 6:**
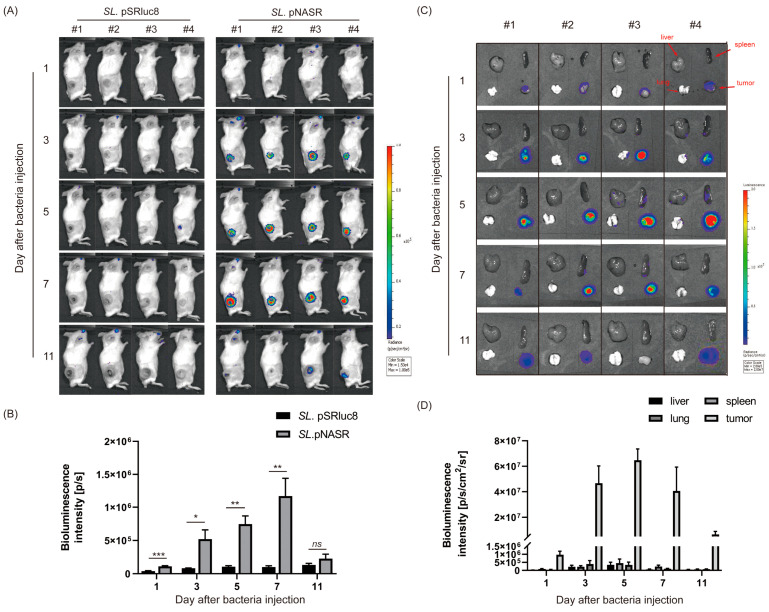
In vivo Rluc8 expression and distribution. (**A**) Bioluminescence images of CT26 tumor-bearing mice 1, 3, 5, 7, and 11 days after injection of 1 × 10^7^ CFUs *SL*. pNASR and *SL*. pSRluc8. (**B**) Quantification of the intensity of bioluminescence signals from the tumor sites. (**C**) Ex vivo bioluminescence images of Rluc8 expression in normal organs (liver, spleen, and lung) and tumor tissues 1, 3, 5, 7, and 11 days after injection of 1 × 10^7^ CFUs *SL*. pNASR. (**D**) Quantification of the intensity of Rluc8 from (**C**). Error bars represent the mean ± SEM (*n* = 4). * *p* < 0.05, ** *p* < 0.01, and *** *p* < 0.001; ns, not significant by Student’s *t*-tests.

The potency of this NO-sensing switch system for in vivo tumor-targeted gene delivery was evaluated (Figure 6). CT26 tumor-bearing mice were intravenously injected with 1 × 10^7^ CFUs of *SL*. pNASR or *SL*. pSRluc8 as control. As expected, tumors in control mice showed no detectable levels of bioluminescence signal, as there was no NO-driven FimE expression (Figure 6A). By contrast, signals were detected in the tumor region on day 3 after *SL*. pNASR injection, with these levels being increased, reaching a maximal level on day 7 (Figure 6A,B). Despite iNOS expression being decreased on days 5 and 7, the Rluc8 intensity increased. These results also confirmed that once the switch system is activated, gene expression is maintained, even after changes in NO generation in the tumor microenvironment. This in vivo result was also consistent with the ex vivo Rluc8 signal in organs or tumor tissues obtained from mice treated with *SL*. pNASR (Figure 6C,D). Beginning 3 days after injection, strong Rluc8 signals were detected in the tumor region, with high tumor-to-normal organ ratios, further confirming that genes delivered by this NO-sensing switch system are tumor-specific.

**Figure 7:**
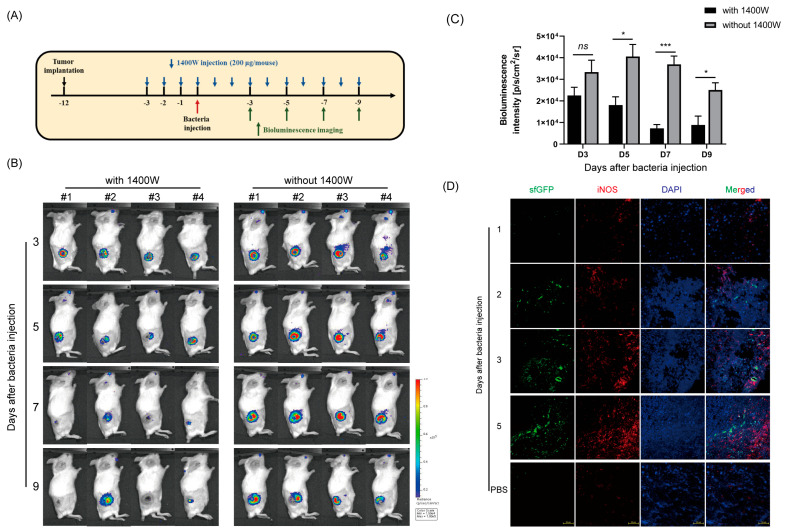
In vivo NO−specific switch activation. (**A**) Scheme of the experiment. CT26 tumor-bearing mice were intraperitoneally administered 1400 W daily, starting 3 days before *SL*. pNASR injection until the end of the experiment. Bioluminescence imaging was performed 3, 5, 7, and 9 days after administration of *SL*. pNASR. (**B**) Bioluminescence images of CT26 tumor-bearing mice in the presence or absence of 1400 W treatment 3, 5, 7, and 9 days after injection of 1 × 10^7^ CFUs *SL*. pNASR. (**C**) Quantification of bioluminescence signals in the tumor tissues. Values represent the mean ± SEM. * *p* < 0.05, ** *p* < 0.01, and *** *p* < 0.001; ns, not significant by Student’s *t*-tests. (**D**) Immunofluorescence staining of iNOS and sfGFP expression in CT-26 tumor tissues on the indicated days after *SL*. pNASGFP injection. Switch activation was indicated by the expression of sfGFP (green), sections were stained with anti-iNOS antibody (red), and nuclei were stained with DAPI (blue). Merged images are also shown (merged). Scale bar, 50 μm. Data are representative of three independent experiments.

To assess whether the NO-sensing gene switch system was activated by NO generated by iNOS expression, CT-26 tumor-bearing mice were intraperitoneally treated with the iNOS inhibitor 1400 W dihydrochloride once daily [41], beginning 3 days before *SL*. pNASR injection until the end of the experiment, and bioluminescence images of the mice were taken after bacterial injection on the indicated days (Figure 7). The signals in the tumor tissues were lower in mice than that were not treated with 1400 W on day 3, with the difference being significant on days 5, 7, and 9 (Figure 7B,C). This finding indicated that this switch system was specifically induced by NO produced by iNOS, a finding confirmed by co-localization of iNOS and GOI expression via immunofluorescence staining. *SL*. pNASGFP was used instead of *SL*. pNASR to visualize gene expression in tumors, with switch activation indicated by the expression of sfGFP. Strong sfGFP signals were detected at sites with increased iNOS expression from tumor sections on days 2, 3, and 5 (Figure 7D). 

These results indicated that the gene switch system described in this study could be specifically activated by NO in tumors after bacterial colonization, and can realize tumor-targeted gene delivery.

## 4. Discussion 

The present study used *SL*. ΔppGpp with a nitric oxide (NO)-sensing switch system to show that elevated NO in tumors generated by upregulated inducible nitric oxide synthase (iNOS) after *S.* Typhimurium infection can induce tumor-targeted gene delivery. Sensing of NO by the switch system led to the expression of a site-specific DNA recombinase, which induced the permanent inversion of a promoter region, resulting in the sustainable expression of a target gene. 

Although tumor-targeting bacteria can reach high tumor/normal organ ratio, the presence of bacterial cells in normal tissue upon systemic administration could damage normal tissues. To reduce non-specific gene expression and off-target effects, efforts have been made to regulate payload expression by placing the gene under the control of a promoter responding to a specific signal given at proper time points, rather than using constitutively promoters [7,42]. These signals can be external chemicals, such as L-arabinose to induce the P_BAD_ promoter [43] and tetracycline that triggers a bidirectional P_Tet_ promoter [44]. Multiple doses of these inducers are required to induce and maintain the continuous expression of the payloads, which have been found to cause side effects in mice [45]. Endogenous signals from unique tumor microenvironments have also been developed, including hypoxia [46] and acidic pH in tumors [47]. Endogenous inducers may overcome the drawbacks associated with exogenous inducers, but changes in the tumor microenvironment during treatment may also affect therapeutic efficacy.

Developments in synthetic biology have enabled the design of more complex gene circuits [48]. A memory circuit, which was designed to record cellular events via permanent changes to DNA caused by recombinase in response to a transient input signal, was used to engineer diagnostics and controllable sustained therapeutic expression [49]. Use of a recombinase-based memory circuit can convert a temporary trigger to a long-term cellular response, overcoming the requirement for continuous stimulation of the inducible promoters.

The present study describes the construction of a NO-activated memory circuit, resulting in long-term NO-driven gene expression carried by *SL*. ΔppGpp. NorR detection of NO was found to lead to the expression of a DNA recombinase, FimE, which catalyzed the permanent and inheritable inversion of a DNA switch, *fimS*, leading to the sustainable expression of the GOI. This switch system was shown to respond to chemical source of NO (DETA/NO, diethylenetriamine/nitric oxide) in a rapid and dose-dependent manner in vitro. Removal of DETA/NO after switch activation did not affect Rluc8 expression in bacterial cells, indicating that the activated switch can be maintained and passaged via replication. In vivo NO-induced tumor target gene delivery was tested by bioluminescence imaging. This switch could be specifically activated in tumors after bacterial colonization, with a high tumor-to-normal organ ratio, and 1400 W inhibition of iNOS activity demonstrated that switch activation was specific to NO generated by iNOS. These results confirmed that NO can be used as an inducer for tumor-specific gene delivery by *S*. Typhimurium.

## 5. Conclusions 

Taken together, our study showed that the increased NO in tumors after *SL*. ΔppGpp colonization can induce tumor-specific gene delivery. The permanent and inheritable switch activation caused by DNA recombinase enabled sustained gene expression in tumors. This NO-sensing switch system could represent a promising strategy for tumor-targeted gene delivery for diagnostic and/or therapeutic purposes.

## Data Availability

The data that support the findings of this study are available from the corresponding author upon reasonable request.

## Supplementary Materials

The following supporting information can be downloaded at: https://www.mdpi.com/article/10.3390/bios13020266/s1, Figure S1: Switch activation percentage under various concentrations of DETA/NO. Figure S2: Time-dependent switch event in bacterial cells with the NO−sensing switch system. Table S1: Plasmids and *SL*. ΔppGpp transformants.
