# Supplementary material for: Genetic Programming by Nitric Oxide-Sensing Gene Switch System in Tumor-Targeting Bacteria
Source: Biosensors (Basel). 2023 Feb 13;13(2):266. doi: 10.3390/bios13020266 (PMC9954711; doi:10.3390/bios13020266)
Supplement: Supplementary file 1 [file biosensors-13-00266-s001.zip › biosensors-2057166-supplementary.pdf]

biosensors

MDPI

Supplementary Materials

Genetic Programming by Nitric Oxide-Sensing Gene Switch System in Tumor-Targeting Bacteria

Yeshan Qin [1,2] Sung-Hwan You [2] Ying Zhang [1,2] Akhil Venu [2] Yeongjin Hong [3,*] and Jung-Joon Min [1,2,4,*]

[1] Department of Molecular Medicine, Chonnam National University Graduate School, 61469 Gwangju, Republic of Korea; 186237@jnu.ac.kr (Y.Q.); 188675@jnu.ac.kr (Y.Z.)
[2] Institute for Molecular Imaging and Theranostics, Hwasun Hospital, Chonnam National University Medical School, 58128 Hwasun, Republic of Korea; yshyoou@naver.com (S.-H. Y); 177894@jnu.ac.kr (A.V.)
[3] Department of Microbiology, Chonnam National University Medical School, 58128 Hwasun, Republic of Korea
[4] Department of Nuclear Medicine, Chonnam National University Medical School, 61469 Gwangju, Republic of Korea
* Correspondence: jjmin@jnu.ac.kr (J.-J.M.); yjhong@jnu.ac.kr (Y.H.); Tel.: +82-61-379-2876 (J.-J.M.); +82-61-379-2746 (Y.H.); Fax: +82-61-379-2875 (J.-J.M.); +82-61-379-2875 (Y.H.)

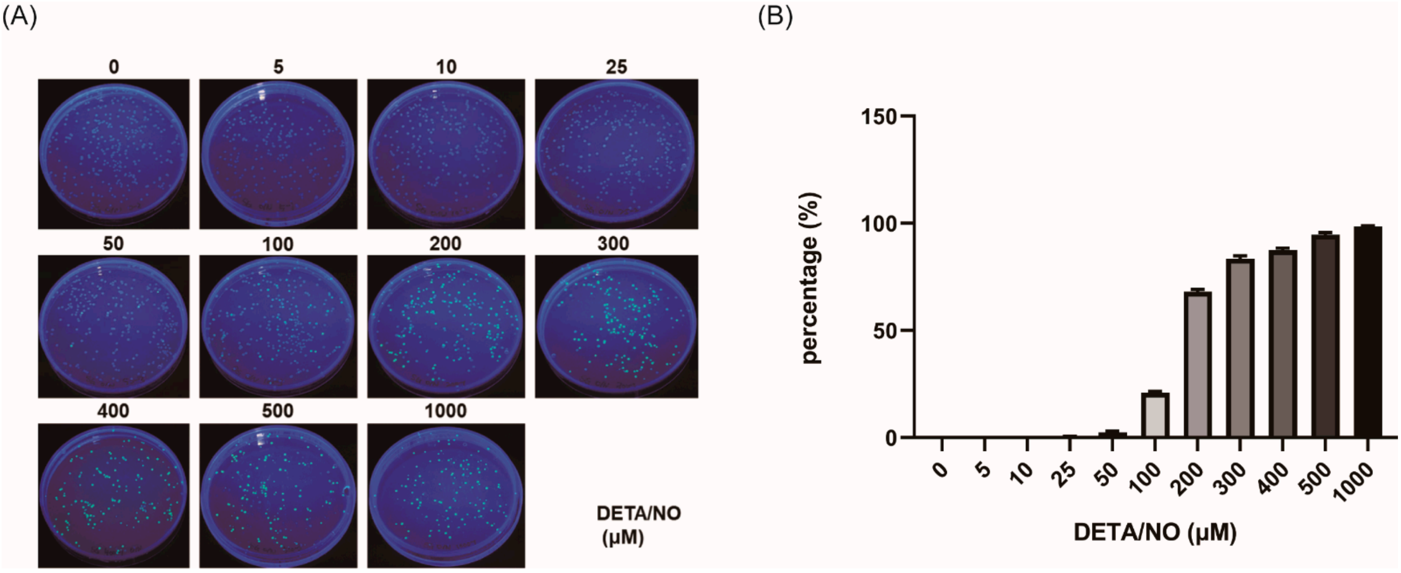

Figure S1. Switch activation percentage under various concentrations of DETA/NO. *SL.* pNASGFP were incubated with the indicated concentrations of DETA/NO for 16 h at 37°C. The bacteria were spread on agar plates containing antibiotics and cultured overnight. (A) Representative UV photos of sfGFP-positive colonies on agar plates. (B) Percentages of activated switching in *SL.* pNASGFP at the indicated concentrations of DETA/NO. Percentages (mean ± SEM) were calculated as the number of sfGFP-positive colonies relative to total colonies. Assays were performed in triplicate.

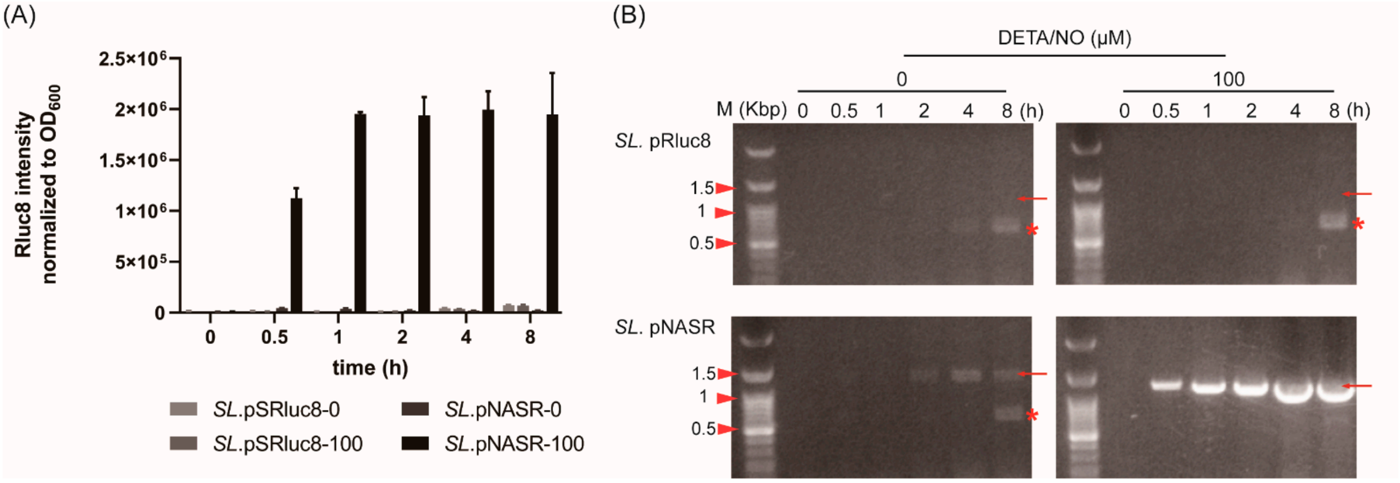

Figure S2. Time-dependent switch event in bacterial cells with the NO−sensing switch system. *SL.* pNASR was cultured for the indicated times in the presence (100 μM) or absence (0 μM) of DETA/NO; *SL.* pSRluc8 was used as the negative control. (A) Rluc8 intensity normalized to number of bacterial cells (OD_{600}). (B) PCR analysis of gene switch events. The switched genes were amplified with ON-state primers (arrows). *, non-specific bands. Results shown as the mean ± SEM of triplicate determinations.

Table S1. Plasmids and *SL*. ΔppGpp transformants.

Plasmid	Constructs	Antibiotic resistance
pFimE	NO inducible FimE expression	Chloramphenicol
pSRluc8	*rluc8* as GOI in *fimS* switch block, with constitutively expressed NorR	Ampicillin
pSGFP	*sfGFP* as GOI in *fimS* switch block, with constitutively expressed NorR	Ampicillin

Strain	Genotype	Purpose
SL. pSRluc8	*SL.* ΔppGpp + pSRluc8	Control
SL. pNASR	*SL.* ΔppGpp + pFimE + pSRluc8	*rluc8* as reporter gene
SL. pNASGFP	*SL.* ΔppGpp + pFimE + pSGFP	*sfGFP* as reporter gene

GOI: gene of interest.

rluc8: *renilla* luciferase variant 8

sfGFP: *super-folder green fluorescent protein*.
